# Disparity and Trends in Secondhand Smoke Exposure among Japanese Employees, Particularly Smokers vs. Non-Smokers

**DOI:** 10.1371/journal.pone.0152096

**Published:** 2016-04-06

**Authors:** Takahiro Tabuchi, Brian Colwell

**Affiliations:** 1 Center for Cancer Control and Statistics, Osaka Medical Center for Cancer and Cardiovascular Diseases, Osaka, Japan; 2 Department of Health Promotion & Community Health Sciences, Texas A&M School of Public Health, College Station, Texas, United States of America; Aichi Cancer Center Research Institute, JAPAN

## Abstract

**Background:**

Monitoring disparities in secondhand smoke (SHS) exposure is important for tailoring smoke-free policies to the needs of different groups. We examined disparity and trends in SHS exposure among both nonsmokers and smokers at Japanese workplaces between 2002 and 2012.

**Methods:**

A total of 32,940 employees in nationally representative, population-based, repeated cross-sectional surveys in 2002, 2007 and 2012 in Japan was analyzed. Adjusted rate ratios for workplace SHS exposure from other people (“everyday” and “everyday or sometimes”) were calculated according to covariates, using log-binomial regression models with survey weights. In this survey, employees who do not smoke at workplace are defined as workplace-nonsmokers; and those smoke at workplace are used as workplace-smokers. SHS exposure for smokers does not involve their own SHS.

**Results:**

While everyday SHS exposure prevalence in workplace-nonsmokers decreased markedly (33.2% to 11.4%), that in workplace-smokers decreased only slightly (63.3% to 55.6%). Workplace-smokers were significantly more likely to report everyday SHS exposure than workplace-nonsmokers, and the degree of association increased over time: compared with the nonsmokers (reference), covariates-adjusted rate ratio (95% confidence interval) for the smokers increased from 1.70 (1.62–1.77) in 2002 to 4.16 (3.79–4.56) in 2012. Similar results were observed for everyday or sometimes SHS exposure. Compared with complete workplace smoking bans, partial and no bans were consistently and significantly associated with high SHS exposure among both nonsmokers and smokers. We also observed disparities in SHS exposure by employee characteristics, such as age group and worksite scale.

**Conclusions:**

Although overall SHS exposure decreased among Japanese employees between 2002 and 2012, the SHS exposure disparity between nonsmokers and smokers widened. Because smokers reported more frequent SHS exposure than nonsmokers, subsequent mortality due to SHS exposure may be higher in smokers than in nonsmokers. This information may be useful for advocating workplace smoke-free policies.

## Introduction

Secondhand smoke (SHS) exposure is a modifiable risk factor for respiratory, cardiovascular, and neoplastic diseases [1]. Worldwide, approximately 35% of nonsmokers have been exposed to SHS at some point [2], with the workplace reported to be a major space where such exposure occurs [3]. To reduce workplace SHS exposure, complete workplace smoking bans have been recommended in the World Health Organization Framework Convention on Tobacco Control (FCTC). However, many countries—including Germany, Switzerland, and Japan [4–8]—have instead implemented partial smoking bans, with the establishment of designated areas where smoking is still allowed. In Japan, complete smoking bans have not been mandated by any Japanese law; the Health Promotion Law allows for partial bans as an option, and the Workplace Smoke-free Guideline recommended a partial rather than a complete ban in 2003 [9–11]. Similarly, the Industrial Safety and Health Act, which asks for appropriate management to prevent SHS exposure at workplaces beginning in 2015, does not mandate a complete smoking ban.

Monitoring SHS exposure disparities and trends is important for helping lawmakers tailor policies to the needs of different groups [3,5,12]. Information on SHS exposure among smokers may help this population recognize the full scope of harm caused by SHS exposure—both to themselves and others—thereby contributing to the establishment of smoke-free societies. However, because most previous studies have targeted nonsmokers alone [1], those assessing SHS exposure disparities between nonsmokers and smokers are scarce [13–15]. Here, we examined the distribution, determinants, and secular trends of SHS exposure among employees of Japanese workplaces, including not only nonsmokers but also smokers.

## Methods

### Data

We used data from the nationally representative, population-based, repeated cross-sectional, "Survey on State of Employees' Health" conducted by the Japanese Ministry of Health, Labour and Welfare (MHLW) in 2002, 2007, and 2012. In these surveys, registered business establishments (worksites) with at least 10 employees (excluding public offices) in Japan were randomly sampled, and a worksite-level questionnaire was sent to the person responsible for "industrial safety and health (*rodo-anzen-eisei*)" at each location. Additionally, data were collected from a representative sample of individual employees randomly sampled from those same worksites which responded to the questionnaire. For the individual data collection, worksites were stratified based on number of employees and randomly selected for the individual survey. Sample sizes and response rates are shown in Table 1. The MHLW calculated the survey weights to represent the total population of Japanese employees, additionally accounting for non-responses and sampling probability [8]. Data were used with permission from the MHLW. The study was reviewed and approved by the Research Ethics Committee of the Osaka Medical Center for Cancer and Cardiovascular Diseases (No.1508119060).

**Table 1:** Study subjects: worksites and individuals sampled.

Survey years	2002	2007	2012
Worksites sampled	12,634	13,609	13,609
Worksites responding (response rate)	9893 (78.3%)	9634 (70.8%)	9283 (69.6%)
Employees (individuals) selected	16,081	17,785	18,075
Employees responding (response rate)	11,707 (72.8%)	11,440 (64.3%)	9915 (56.7%)
Number of worksites with individual employee responses	1658	1145	1033

### Variables

Questions regarding the frequency of SHS exposure asked: "*How often are you exposed to other people's cigarette smoke in your workplace*?" (almost every day, sometimes, or never). Thus, SHS exposure for smokers does not involve their own SHS.

The following variables were used as potential determinants of SHS exposure in the analysis: smoking status, sex, age group (≤29, 30–39, 40–49, 50–59 or ≥60 years), employment category (regular employee or other, including part-time workers), worksite scale based on number of employees (10–29, 30–49, 50–99, 100–299, 300–999 or ≥1000 employees), and workplace smoke-free policy (complete, partial, or no ban [8,9]). A workplace-smoker was defined as a person who smoked cigarettes regularly at the workplace (yes or no). In this study, employees who do not smoke at the workplace were defined as workplace-nonsmokers even if they smoked at other site, because only workplace smoking behavior was queried.

### Statistical analysis

The prevalence of SHS exposure was calculated using survey weights for generalizability to the whole country. The rate ratios (RRs) and 95% confidence intervals (CIs) for SHS exposure were calculated. First, to examine the determinants of SHS exposure, stratified data by smoking status were used, because SHS exposure may be different according to smoking status. Second, to observe disparities and trends in SHS exposure across characteristics including smoking status, total data including both workplace-smokers and workplace-nonsmokers were used. Log-binomial regression models were used because the outcome was not rare (more than 10%) [16]. In some instances, the models did not converge, so we used log-Poisson models, which provide consistent estimates of RRs [17]. Stabilized weights (weight divided by the mean of the weight) were used in regressions to estimate acceptable 95% CIs [8,18], as using non-stabilized weights resulted in extremely narrow 95% CIs (e.g. 0.981–0.984). Subjects with missing information on covariates were excluded from the regression analyses in a listwise manner. All analyses were performed using SAS version 9.3 (SAS Institute, Cary, NC, USA). Probability values for statistical tests were two-tailed, and p <0.05 was considered statistically significant.

## Results

Basic characteristics of study subjects are shown in Table 2 (unweighted values: S1 Table). The total weighted number, approximating total employee distribution in Japan, was 32,482,081 in 2002, 29,322,435 in 2007, and 36,018,201 in 2012, although the unweighted number was 32,940 in total (11,707 in 2002, 11,340 in 2007 and 9893 in 2012). Smoking prevalence (at the workplace) decreased from 39.3% in 2002 to 26.9% in 2012. The proportion of employees in workplaces with complete smoking bans increased from 2.1% in 2002 to 35.6% in 2012, while the proportion in workplaces with partial bans increased from 64.9% in 2002 to 69.9% in 2007, but then decreased to 49.8% in 2012. As for no bans, the proportion decreased from 33.0% in 2002 to 14.6% in 2012.

**Table 2:** Basic characteristics of study subjects in percent (weighted results).

Characteristics	2002	2007	2012	Total
Weighted number	32,482,081	29,322,435	36,018,201	97,822,717
Smoking status				
Nonsmoker at the workplace	60.7	65.6	73.1	66.7
Smoker at the workplace	39.3	34.4	26.9	33.3
Sex				
Men	61.9	59.3	55.5	58.8
Women	38.1	40.7	44.5	41.2
Age group				
less than 30 years	22.9	21.1	18.8	20.9
30–39 years	25.0	26.9	28.3	26.8
40–49 years	25.5	23.9	25.4	25.0
50–59 years	22.6	22.6	19.4	21.4
60 years or more	4.1	5.5	8.1	6.0
Employment category				
Regular employee	83.0	77.0	74.0	77.9
Others including part-time worker	17.0	23.0	26.0	22.1
Worksite scale (employee number)				
10–29	36.0	36.6	33.7	35.3
30–49	14.3	13.6	11.6	13.1
50–99	15.7	16.8	14.4	15.6
100–299	19.4	17.6	21.2	19.5
300–999	9.9	10.5	11.0	10.5
1000 or more	4.8	5.0	8.1	6.1
Workplace smoking ban status				
Complete ban	2.1	9.9	35.6	16.8
Partial ban	64.9	69.9	49.8	60.8
No ban	33.0	20.2	14.6	22.4

The unweighted number of missing values was 239 for employment category in 2012 (see S1 Table). No other variables had missing values.

To examine determinants of SHS exposure, prevalence of and covariates-adjusted RRs for workplace SHS exposure stratified by smoking status are shown in Table 3 (unweighted values: S2 Table). Everyday SHS exposure prevalence was 21.2% in workplace-nonsmokers and 60.3% in workplace-smokers, while everyday or sometimes SHS exposure prevalence was 57.1% and 83.6%, respectively. For all eight categories (2x2x2 = 8; i.e., [workplace-nonsmokers and workplace-smokers] x [everyday SHS and everyday or sometimes SHS] x [weighted result and unweighted result]), compared with complete workplace smoking bans, partial and no bans were significantly associated with high SHS exposure, although the degree of the association was larger among nonsmokers than smokers. Similarly for all eight categories, younger age was associated with higher SHS exposure. Further, we also observed disparities in SHS exposure by employee characteristics such as sex, employment category and worksite scale, although there were some exceptions.

**Table 3:** Trends in prevalence and rate ratio for workplace SHS exposure from other people among employees according to characteristic, stratified by smoking status. Combined all years of 2002, 2007 and 2012 (weighted results).

	Nonsmokers at the workplace				Smokers at the workplace			
Characteristics	Everyday SHS exposure (%)	Rate ratio^a^(95% CI)	Everyday or sometimes SHS exposure (%)	Rate ratio^a^(95% CI)	Everyday SHS exposure (%)	Rate ratio^a^(95% CI)	Everyday or sometimes SHS exposure (%)	Rate ratio^a^(95% CI)
Total	21.2	NA	57.1	NA	60.3	NA	83.6	NA
Survey year								
2002	33.2	1 (reference)	72.9	1 (reference)	63.3	1 (reference)	86.3	1 (reference)
2007	19.2	**0.68 (0.65, 0.72)**	56.4	**0.86 (0.84, 0.88)**	60.0	0.98 (0.94, 1.01)	83.8	1.00 (0.98, 1.01)
2012	11.4	**0.54 (0.50, 0.58)**	42.2	**0.75 (0.73, 0.77)**	55.6	0.97 (0.93, 1.01)	78.7	0.98 (0.96, 1.001)
Sex								
Men	24.0	1 (reference)	63.3	1 (reference)	60.7	1 (reference)	84.1	1 (reference)
Women	18.8	**0.79 (0.75, 0.83)**	51.7	**0.89 (0.87, 0.91)**	58.4	0.96 (0.92, 1.0002)	81.4	0.99 (0.97, 1.02)
Age group								
less than 30 years	24.7	1 (reference)	61.7	1 (reference)	68.9	1 (reference)	88.7	1 (reference)
30–39 years	20.0	**0.87 (0.82, 0.94)**	57.2	**0.97 (0.95, 0.99)**	64.3	**0.94 (0.91, 0.98)**	86.9	0.99 (0.97, 1.01)
40–49 years	19.2	**0.80 (0.75, 0.86)**	54.3	**0.93 (0.90, 0.95)**	57.6	**0.84 (0.81, 0.88)**	82.1	**0.94 (0.92, 0.96)**
50–59 years	22.8	**0.89 (0.83, 0.95)**	57.8	**0.96 (0.93, 0.98)**	53.2	**0.78 (0.74, 0.82)**	77.3	**0.90 (0.88, 0.92)**
60 years or more	16.2	**0.69 (0.61, 0.77)**	49.8	**0.86 (0.82, 0.90)**	37.4	**0.57 (0.50, 0.65)**	73.6	**0.86 (0.81, 0.91)**
Employment category								
Regular employee	22.4	**1.11 (1.05, 1.19)**	59.9	**1.13 (1.10, 1.16)**	60.9	1.04 (0.99, 1.09)	84.3	**1.05 (1.02, 1.08)**
Others including part-time worker	18.3	1 (reference)	50.2	1 (reference)	55.7	1 (reference)	79.0	1 (reference)
Worksite scale (employee number)								
10–29	24.7	**1.59 (1.37, 1.84)**	60.7	**1.26 (1.19, 1.34)**	59.9	**0.85 (0.80, 0.91)**	83.4	0.99 (0.95, 1.03)
30–49	26.3	**1.63 (1.40, 1.90)**	65.8	**1.30 (1.23, 1.38)**	59.0	**0.87 (0.82, 0.94)**	84.3	1.00 (0.96, 1.04)
50–99	22.5	**1.54 (1.32, 1.79)**	57.7	**1.23 (1.16, 1.31)**	59.7	**0.88 (0.82, 0.94)**	84.8	1.02 (0.97, 1.06)
100–299	17.7	**1.19 (1.02, 1.39)**	55.1	**1.15 (1.08, 1.22)**	60.7	**0.91 (0.85, 0.97)**	83.9	1.02 (0.98, 1.06)
300–999	13.9	1.08 (0.91, 1.28)	45.7	1.02 (0.95, 1.09)	63.6	0.97 (0.90, 1.05)	82.4	1.01 (0.96, 1.05)
1000 or more	12.5	1 (reference)	44.3	1 (reference)	65.1	1 (reference)	81.8	1 (reference)
Workplace smoking ban status								
Complete ban	4.8	1 (reference)	27.6	1 (reference)	42.2	1 (reference)	66.9	1 (reference)
Partial ban	20.7	**3.34 (2.88, 3.87)**	60.1	**1.93 (1.83, 2.04)**	59.3	**1.31 (1.21, 1.42)**	83.0	**1.21 (1.15, 1.27)**
No ban	37.2	**5.28 (4.55, 6.14)**	74.1	**2.21 (2.09, 2.34)**	68.1	**1.55 (1.43, 1.68)**	90.1	**1.31 (1.25, 1.38)**

CI, confidence interval; NA, not applicable; SHS, secondhand smoke.

^a^Adjusted for listed all variables.

Boldface indicates statistical significance (p < 0.05).

The trends in prevalence of workplace SHS exposure from 2002 to 2012 were also shown in Table 3 (rows of "survey year"). Everyday SHS exposure prevalence in workplace-nonsmokers decreased from 33.2% in 2002 to 11.4% in 2012, while prevalence in workplace-smokers decreased from 63.3% in 2002 to 55.6% in 2012, a smaller range of decrease than in nonsmokers.

To observe trend in SHS exposure, particularly smokers vs. non-smokers, Table 4 shows the trends in prevalence and RR for everyday SHS exposure from 2002 to 2012 (unweighted values: S3 Table). Overall prevalence (including both nonsmokers and smokers at workplace) decreased from 45.0% in 2002 to 23.3% in 2012. Workplace-smokers were significantly more likely to report everyday SHS exposure than workplace-nonsmokers, and the degree of the association increased over time, with RR (95% CI) increasing from 1.70 (1.62–1.77) in 2002 to 4.16 (3.79–4.56) in 2012.

**Table 4:** Trends in prevalence and rate ratio for everyday workplace SHS exposure from other people among employees according to characteristics (weighted results).

	2002		2007		2012	
	Everyday SHS exposure (%)	Rate ratio^a^ (95% CI)	Everyday SHS exposure (%)	Rate ratio^a^ (95% CI)	Everyday SHS exposure (%)	Rate ratio^a^ (95% CI)
**Total**	45.0	NA	33.2	NA	23.3	NA
**Smoking status**						
Nonsmoker at the workplace	33.2	1 (reference)	19.2	1 (reference)	11.4	1 (reference)
Smoker at the workplace	63.3	**1.70 (1.62, 1.77)**	60.0	**2.69 (2.53, 2.85)**	55.6	**4.16 (3.79, 4.56)**
**Sex**						
Men	51.7	1 (reference)	40.8	1 (reference)	29.3	1 (reference)
Women	34.0	**0.87 (0.83, 0.92)**	22.1	**0.89 (0.83, 0.94)**	15.9	**0.86 (0.78, 0.96)**
**Age group**						
Less than 30 years	47.9	1 (reference)	34.1	1 (reference)	32.8	1 (reference)
30–39 years	48.1	**0.93 (0.89, 0.97)**	36.6	1.05 (0.99, 1.11)	25.2	**0.76 (0.68, 0.85)**
40–49 years	41.7	**0.83 (0.79, 0.87)**	34.5	**0.91 (0.86, 0.97)**	21.8	**0.76 (0.67, 0.85)**
50–59 years	45.2	**0.91 (0.87, 0.96)**	29.0	**0.82 (0.76, 0.88)**	17.9	**0.63 (0.55, 0.72)**
60 years or more	28.9	**0.64 (0.55, 0.73)**	24.9	**0.83 (0.73, 0.95)**	12.2	**0.45 (0.36, 0.56)**
**Employment category**						
Regular employee	47.4	**1.21 (1.13, 1.29)**	35.9	1.00 (0.93, 1.08)	23.9	0.91 (0.82, 1.02)
Others, including part-time worker	33.5	1 (reference)	24.1	1 (reference)	21.8	1 (reference)
**Worksite scale (number of employees)**						
10–29	46.9	0.97 (0.88, 1.07)	37.6	**0.89 (0.81, 0.99)**	27.0	**1.25 (1.03, 1.51)**
30–49	47.7	1.03 (0.93, 1.14)	34.0	**0.88 (0.79, 0.98)**	26.9	**1.30 (1.06, 1.60)**
50–99	46.4	1.00 (0.90, 1.11)	31.9	0.92 (0.82, 1.02)	22.0	1.14 (0.93, 1.40)
100–299	42.1	1.00 (0.90, 1.10)	29.1	**0.80 (0.71, 0.90)**	22.3	**1.22 (1.00, 1.49)**
300–999	39.6	1.01 (0.91, 1.14)	25.4	**0.84 (0.74, 0.96)**	17.3	1.00 (0.79, 1.25)
1000 or more	41.0	1 (reference)	34.8	1 (reference)	16.0	1 (reference)
**Workplace smoking ban status**						
Complete ban	13.1	1 (reference)	7.3	1 (reference)	14.0	1 (reference)
Partial ban	40.7	**2.71 (1.97, 3.72)**	33.2	**3.40 (2.75, 4.19)**	24.2	**1.51 (1.36, 1.69)**
No ban	55.4	**3.45 (2.51, 4.76)**	46.0	**4.01 (3.24, 4.96)**	43.0	**2.38 (2.10, 2.69)**

CI, confidence interval; NA, not applicable; SHS, secondhand smoke

^a^Adjusted for all listed variables

Boldface indicates statistical significance (p <0.05).

Similarly, Table 5 shows the trends in prevalence and RR for everyday or sometimes SHS exposure from 2002 to 2012 (unweighted values: S4 Table). Overall exposure prevalence decreased from 78.2% in 2002 to 52.0% in 2012. Similar to findings with everyday SHS exposure, workplace-smokers reported significantly higher SHS exposure than workplace-nonsmokers, and the degree of association increased over time, with RR (95% CI) increasing from 1.09 (1.07–1.12) in 2002 to 1.60 (1.51–1.70) in 2012, although the magnitudes of RR were smaller than those of everyday SHS exposure.

**Table 5:** Trends in prevalence and rate ratio for workplace SHS exposure from other people (everyday or sometimes) among employees according to characteristics (weighted results).

	2002		2007		2012	
	Everyday or sometimes SHS exposure (%)	Rate ratio^a^ (95% CI)	Everyday or sometimes SHS exposure (%)	Rate ratio^a^ (95% CI)	Everyday or sometimes SHS exposure (%)	Rate ratio^a^ (95% CI)
**Total**	78.2	NA	65.8	NA	52.0	NA
**Smoking status**						
Nonsmoker at the workplace	72.9	1 (reference)	56.4	1 (reference)	42.2	1 (reference)
Smoker at the workplace	86.3	**1.09 (1.07, 1.12)**	83.8	**1.30 (1.27, 1.34)**	78.7	**1.60 (1.51, 1.70)**
**Sex**						
Men	82.7	1 (reference)	73.6	1 (reference)	60.5	1 (reference)
Women	70.7	**0.96 (0.94, 0.98)**	54.5	**0.91 (0.88, 0.94)**	41.6	**0.82 (0.77, 0.88)**
**Age group**						
Less than 30 years	79.8	1 (reference)	67.1	1 (reference)	61.7	1 (reference)
30–39 years	79.7	0.99 (0.96, 1.01)	68.2	1.00 (0.98, 1.02)	56.9	**0.93 (0.87, 1.01)**
40–49 years	78.2	0.98 (0.96, 1.00)	64.6	**0.92 (0.90, 0.95)**	47.9	**0.81 (0.75, 0.88)**
50–59 years	77.5	**0.98 (0.95, 1.00)**	64.8	**0.96 (0.94, 0.99)**	43.2	**0.73 (0.66, 0.80)**
60 years or more	62.6	**0.87 (0.81, 0.93)**	59.0	**0.86 (0.81, 0.92)**	46.7	**0.80 (0.71, 0.91)**
**Employment category**						
Regular employee	81.4	**1.26 (1.21, 1.30)**	68.1	0.99 (0.96, 1.03)	53.5	1.00 (0.93, 1.08)
Others, including part-time worker	62.5	1 (reference)	58.0	1 (reference)	49.0	1 (reference)
**Worksite scale (number of employees)**						
10–29	78.2	**1.07 (1.01, 1.12)**	69.9	**1.10 (1.03, 1.18)**	57.2	**1.29 (1.14, 1.46)**
30–49	84.0	**1.12 (1.06, 1.18)**	67.5	1.05 (0.98, 1.13)	60.9	**1.36 (1.19, 1.55)**
50–99	78.2	**1.09 (1.03, 1.15)**	66.8	**1.11 (1.04, 1.19)**	51.4	**1.18 (1.04, 1.35)**
100–299	77.4	**1.08 (1.03, 1.15)**	64.6	**1.08 (1.01, 1.16)**	49.9	1.13 (1.00, 1.28)
300–999	73.8	**1.08 (1.02, 1.14)**	52.9	0.96 (0.89, 1.04)	39.9	0.91 (0.79, 1.05)
1000 or more	72.3	1 (reference)	60.4	1 (reference)	41.4	1 (reference)
**Workplace smoking ban status**						
Complete ban	34.2	1 (reference)	30.3	1 (reference)	37.3	1 (reference)
Partial ban	76.5	**2.19 (1.84, 2.60)**	66.5	**2.02 (1.85, 2.22)**	56.9	**1.49 (1.39, 1.59)**
No ban	84.2	**2.38 (2.00, 2.83)**	81.1	**2.28 (2.08, 2.50)**	71.5	**1.71 (1.57, 1.86)**

CI, confidence interval; NA, not applicable; SHS, secondhand smoke

^a^Adjusted for all listed variables

Boldface indicates statistical significance (p <0.05).

## Discussion

While the prevalence of workplace SHS exposure decreased over time, the disparity in exposure between nonsmokers and smokers widened from 2002 to 2012 in Japan. The establishment of partial smoking ban policies in the workplace, separating smokers from nonsmokers, may have concentrated SHS exposure in smokers. Covariates-adjusted RR for everyday SHS exposure increased from 1.70 in 2002 to 4.16 in 2012 among workplace-smokers compared with workplace-nonsmokers, and while everyday SHS exposure decreased markedly to 11.4% in 2012 from 33.2% in 2002 among workplace-nonsmokers, it decreased only slightly—to 55.6% in 2012 from 63.3% in 2002—among workplace-smokers (Table 3). Taken together, our findings suggest that, in addition to being exposed to mainstream cigarette smoke, smokers also experience more frequent exposure to SHS than nonsmokers through greater exposure to fellow smokers.

Although smokers may not consider SHS to be harmful to themselves [19], the harm of SHS exposure in smokers nevertheless merits consideration. In 2004, 603,000 nonsmokers' deaths worldwide were attributable to SHS exposure, comprising approximately 1.0% of worldwide mortality [2]. Because workplace-smokers reported more frequent SHS exposure than workplace-nonsmokers over time, our findings suggest that SHS exposure may have killed a higher ratio of smokers than nonsmokers. However, discriminating the effects of SHS from those of mainstream smoke will be difficult and is outside of the scope of this study, although some studies have challenged the estimation of the effect of SHS on smokers [13–15].

Secondhand smoke exposure occurs in designated smoking rooms or in smoking-allowed spaces (i.e. partial or no bans). While SHS exposure among employees of companies with partial bans decreased from 2007 to 2012, partial bans remained the major avenue of worksite tobacco control efforts, likely because the tobacco industry has strongly lobbied for and promoted separation of smoking areas, with media campaigns to sidetrack efforts to make workplaces entirely smoke-free [20–22]. Because of these efforts, even nonsmokers show more support for partial bans than complete bans in Japan (54% vs. 35%) [10]. The preponderance of partial bans may also be due to the fact that complete smoking bans to prevent SHS exposure have not been mandated by any Japanese law. Neither the Health Promotion Law, the Workplace Smoke-free Guideline nor the Industrial Safety and Health Act (changed in 2015) mandates a complete smoking ban.

Perhaps predictably, the current study also found that partial bans more often led to increased SHS exposure among both workplace-nonsmokers and workplace-smokers than complete bans. Levels of SHS exposure in and adjacent to designated smoking rooms have been reported to be high [23,24], indicating that smoking rooms are not providing safe air quality for employees. As such, the harm of SHS exposure in and around smoking rooms cannot be ignored.

However, complete smoking bans have significantly decreased SHS exposure among Japanese employees (Tables 3–5). Despite the lack of any penalty under Japanese smoke-free policies for not having smoke-free facilities, the proportion of employees working in companies with complete bans increased over 30% in the past decade. The present study confirmed the previous finding that complete smoking bans were significantly associated with lower levels of SHS exposure than partial or no bans over time [25]. We previously found that implementing complete bans in the workplace instead of partial bans decreased the proportion of smoking employees and reports of SHS-related discomfort/ill-health compared to Japanese employees working in companies with no ban [8].

These present and previous findings suggest that protecting not only nonsmokers but also smokers from the harms of SHS exposure will require substantial effort to change public opinion in favor of national, complete smoke-free legislation with appropriate penalties for violation [12,26]. However, complete bans are not a silver bullet solution; indeed, workplace-nonsmokers still experienced 27.6% prevalence of everyday or sometimes SHS exposure in worksites with complete bans during 2002–2012 (Table 3), possibly due to low compliance with smoke-free policies [27] and inappropriate placement of outdoor smoking spaces close to doors or pathways. A key recommendation would be that policy-makers enforce complete smoke-free legislation with penalties to improve compliance and reduce SHS exposure.

Disparities in SHS exposure were also noted with gender, age, and worksite scale. Men, age <40 years, and small worksite scale employees were more likely to report SHS exposure in 2012 after adjustments for covariates than women, subjects aged ≥40 years, and employees at companies with 300 or more workers (Tables 4 and 5), although a difference between workplace-nonsmokers and workplace-smokers was observed especially for worksite scale. Small worksite scale was positively associated with everyday SHS exposure among workplace-nonsmokers, but it was negatively associated with everyday SHS exposure among workplace-smokers. Therefore, it seems that nonsmokers are not protected from SHS especially in small scale workplace, and smokers expose each other to SHS (for example, in a smoking room) especially in large scale workplace. The Japanese health promotion strategy, Health Japan 21 (second version), prioritizes reduction in smoking prevalence and health inequality (including smoking inequality) [28]. From a health inequality perspective, complete smoking bans are necessary in the workplace to protect all employees—including both nonsmokers and smokers—from the harm of cigarette smoke.

### Limitations

Several limitations to the present study warrant mention. First, self-reported SHS exposure was used as the variable of interest, while previous studies have shown that self-reported SHS exposure correlates well with biomarker concentrations [29,30]. In addition, nonsmokers and smokers who did not smoke in workplaces were both coded as nonsmokers in the workplace, because only smoking status in the workplace was available. Smokers who did not smoke at their workplace might have reported SHS exposure differently from nonsmokers, although we could not discriminate them. Second, we could not restrict our sample to those who worked indoors. Because employees who mainly work outdoors or in cars were included in the analysis, their smoking behavior might not be influenced by the workplace smoke-free policy, possibly leading to underestimation of the results with respect to the policy. Third, although weighting to adjust for non-participation may have mitigated the effects of lower response rates over time, survey weights might widen an underlying bias in an unknown direction. However, given the lack of any marked difference between weighted and un-weighted results except with respect to worksite scale, our findings appear to be robust. Despite these limitations, this study has strengths with its large sample size and generalizability for estimating national population impact.

### Conclusions

Although SHS exposure decreased among Japanese employees overall, the exposure disparity between nonsmokers and smokers has widened from 2002 to 2012. From a health inequality perspective, the current study rediscovered smokers as a high-risk population for SHS exposure in addition to mainstream smoke [31]. Smokers may be a little-recognized high-risk population for SHS exposure. Our findings may therefore be useful in advocating workplace smoke-free policies that will benefit both smokers and nonsmokers.

## Supporting Information

S1 Table Basic characteristics of study subjects (total = 32,940; unweighted). (DOCX)

S2 Table Trends in prevalence and rate ratio for workplace SHS exposure from other people among employees according to characteristic, stratified by smoking status. Combined all years of 2002, 2007 and 2012 (unweighted results).
(DOCX)

S3 Table Trends in prevalence and rate ratio for everyday workplace SHS exposure among employees according to characteristics (unweighted results). (DOCX)

S4 Table Trends in prevalence and rate ratio for workplace SHS exposure (everyday or sometimes) among employees according to characteristics (unweighted results). (DOCX)
