# Supplementary material for: Disparity and Trends in Secondhand Smoke Exposure among Japanese Employees, Particularly Smokers vs. Non-Smokers
Source: PLoS One. 2016 Apr 6;11(4):e0152096. doi: 10.1371/journal.pone.0152096 (PMC4822844; doi:10.1371/journal.pone.0152096)
Supplement: S1 Table — (DOCX) [file pone.0152096.s001.docx]

**Table S1. Basic characteristics of study subjects (total =32,940; unweighted)**

|  | 2002 | |  | 2007 | |  | 2012 | |  | Total | |
| --- | --- | --- | --- | --- | --- | --- | --- | --- | --- | --- | --- |
| Characteristics | N | % |  | N | % |  | N | % |  | N | % |
| Total subject | 11707 |  |  | 11340 |  |  | 9893 |  |  | 32940 |  |
| Smoking status |  |  |  |  |  |  |  |  |  |  |  |
| Nonsmoker (at workplace) | 7276 | 62.2 |  | 7861 | 69.3 |  | 7174 | 72.5 |  | 22311 | 67.7 |
| Smoker (at workplace) | 4431 | 37.9 |  | 3479 | 30.7 |  | 2719 | 27.5 |  | 10629 | 32.3 |
| Sex |  |  |  |  |  |  |  |  |  |  |  |
| Men | 7647 | 65.3 |  | 7071 | 62.4 |  | 6343 | 64.1 |  | 21061 | 63.9 |
| Women | 4060 | 34.7 |  | 4269 | 37.7 |  | 3550 | 35.9 |  | 11879 | 36.1 |
| Age group |  |  |  |  |  |  |  |  |  |  |  |
| Less than 30 years | 2465 | 21.1 |  | 2133 | 18.8 |  | 1748 | 17.7 |  | 6346 | 19.3 |
| 30-39 years | 3198 | 27.3 |  | 3325 | 29.3 |  | 2717 | 27.5 |  | 9240 | 28.1 |
| 40-49 years | 2897 | 24.8 |  | 2860 | 25.2 |  | 2822 | 28.5 |  | 8579 | 26.0 |
| 50-59 years | 2670 | 22.8 |  | 2458 | 21.7 |  | 1951 | 19.7 |  | 7079 | 21.5 |
| 60 years or more | 477 | 4.1 |  | 564 | 5.0 |  | 655 | 6.6 |  | 1696 | 5.1 |
| Employment category |  |  |  |  |  |  |  |  |  |  |  |
| Regular employee | 10203 | 87.2 |  | 9457 | 83.4 |  | 7966 | 82.5 |  | 27626 | 84.5 |
| Others, including part-time worker | 1504 | 12.9 |  | 1883 | 16.6 |  | 1688 | 17.5 |  | 5075 | 15.5 |
| Worksite scale (employee number) |  |  |  |  |  |  |  |  |  |  |  |
| 10-29 | 1485 | 12.7 |  | 1628 | 14.4 |  | 1731 | 17.5 |  | 4844 | 14.7 |
| 30-49 | 1483 | 12.7 |  | 1346 | 11.9 |  | 1163 | 11.8 |  | 3992 | 12.1 |
| 50-99 | 1746 | 14.9 |  | 1740 | 15.3 |  | 1378 | 13.9 |  | 4864 | 14.8 |
| 100-299 | 2380 | 20.3 |  | 2364 | 20.9 |  | 1820 | 18.4 |  | 6564 | 19.9 |
| 300-999 | 3403 | 29.1 |  | 2953 | 26.0 |  | 2636 | 26.7 |  | 8992 | 27.3 |
| 1000 or more | 1210 | 10.3 |  | 1309 | 11.5 |  | 1165 | 11.8 |  | 3684 | 11.2 |
| Workplace smoking ban status |  |  |  |  |  |  |  |  |  |  |  |
| Complete ban | 213 | 1.8 |  | 1012 | 8.9 |  | 2801 | 28.3 |  | 4026 | 12.2 |
| Partial ban | 9076 | 77.5 |  | 9174 | 80.9 |  | 6168 | 62.4 |  | 24418 | 74.1 |
| No ban | 2418 | 20.7 |  | 1154 | 10.2 |  | 924 | 9.3 |  | 4496 | 13.6 |

The number of missing values was 239 for employment category in 2012. No other variables had missing values.
