# Supplementary material for: Disparity and Trends in Secondhand Smoke Exposure among Japanese Employees, Particularly Smokers vs. Non-Smokers
Source: PLoS One. 2016 Apr 6;11(4):e0152096. doi: 10.1371/journal.pone.0152096 (PMC4822844; doi:10.1371/journal.pone.0152096)
Supplement: S2 Table — Combined all years of 2002, 2007 and 2012 (unweighted results). (DOCX) [file pone.0152096.s002.docx]

**Table S2. Trends in prevalence and rate ratio for workplace SHS exposure from other people among employees according to characteristic, stratified by smoking status. Combined all years of 2002, 2007 and 2012 (unweighted results)**

|  | Nonsmokers | | | |  | Smokers | | | |
| --- | --- | --- | --- | --- | --- | --- | --- | --- | --- |
| Characteristics | *Everyday SHS exposure (%)* | Rate ratio^a^ (95% CI) | *Everyday or sometimes SHS exposure (%)* | Rate ratio^a^ (95% CI) |  | *Everyday SHS exposure (%)* | Rate ratio^a^ (95% CI) | *Everyday or sometimes SHS exposure (%)* | Rate ratio^a^ (95% CI) |
| Total | 18.3 | NA | 45.8 | NA |  | 59.3 | NA | 82.1 | NA |
| Survey year |  |  |  |  |  |  |  |  |  |
| 2002 | 30.2 | 1 (reference) | 71.4 | 1 (reference) |  | 63.3 | 1 (reference) | 86.4 | 1 (reference) |
| 2007 | 15.3 | **0.59 (0.55, 0.63)** | 51.5 | **0.78 (0.76, 0.80)** |  | 58.6 | **0.95 (0.92, 0.99)** | 81.2 | **0.96 (0.94, 0.98)** |
| 2012 | 9.5 | **0.42 (0.39, 0.46)** | 39.6 | **0.66 (0.64, 0.68)** |  | 53.6 | **0.92 (0.88, 0.96)** | 76.1 | **0.93 (0.90, 0.95)** |
| Sex |  |  |  |  |  |  |  |  |  |
| Men | 19.4 | 1 (reference) | 58.0 | 1 (reference) |  | 59.6 | 1 (reference) | 82.2 | 1 (reference) |
| Women | 17.1 | **0.85 (0.80, 0.90)** | 49.9 | **0.90 (0.88, 0.92)** |  | 57.1 | 0.95 (0.91, 1.004) | 80.9 | 0.99 (0.96, 1.01) |
| Age group |  |  |  |  |  |  |  |  |  |
| less than 30 years | 23.8 | 1 (reference) | 59.7 | 1 (reference) |  | 68.8 | 1 (reference) | 88.3 | 1 (reference) |
| 30-39 years | 18.1 | **0.79 (0.74, 0.85)** | 54.8 | **0.93 (0.91, 0.96)** |  | 61.3 | **0.90 (0.86, 0.93)** | 82.8 | **0.95 (0.93, 0.97)** |
| 40-49 years | 15.8 | **0.69 (0.64, 0.75)** | 50.6 | **0.89 (0.86, 0.92)** |  | 57.1 | **0.84 (0.80, 0.88)** | 80.7 | **0.94 (0.91, 0.96)** |
| 50-59 years | 17.5 | **0.69 (0.64, 0.75)** | 53.7 | **0.91 (0.88, 0.93)** |  | 54.3 | **0.79 (0.75, 0.83)** | 78.8 | **0.90 (0.88, 0.93)** |
| 60 years or more | 14.1 | **0.59 (0.51, 0.68)** | 49.3 | **0.87 (0.82, 0.92)** |  | 44.0 | **0.66 (0.59, 0.74)** | 76.2 | **0.88 (0.84, 0.93)** |
| Employment category |  |  |  |  |  |  |  |  |  |
| Regular employee | 19.2 | **1.14 (1.05, 1.24)** | 43.7 | **1.15 (1.11, 1.19)** |  | 59.8 | 1.04 (0.98, 1.10) | 82.4 | 1.01 (0.98, 1.05) |
| Others including part-time worker | 14.7 | 1 (reference) | 54.6 | 1 (reference) |  | 54.1 | 1 (reference) | 79.6 | 1 (reference) |
| Worksite scale (employee number) |  |  |  |  |  |  |  |  |  |
| 10-29 | 22.5 | **1.62 (1.44, 1.83)** | 59.3 | **1.32 (1.26, 1.39)** |  | 54.3 | **0.89 (0.83, 0.95)** | 82.5 | **1.06 (1.02, 1.11)** |
| 30-49 | 22.0 | **1.53 (1.35, 1.73)** | 60.9 | **1.32 (1.26, 1.39)** |  | 59.7 | 0.97 (0.91, 1.04) | 83.6 | **1.06 (1.02, 1.11)** |
| 50-99 | 21.5 | **1.58 (1.41, 1.78)** | 58.2 | **1.29 (1.23, 1.36)** |  | 58.9 | 0.97 (0.91, 1.03) | 83.1 | **1.07 (1.03, 1.11)** |
| 100-299 | 18.2 | **1.38 (1.23, 1.56)** | 56.0 | **1.25 (1.19, 1.31)** |  | 58.6 | 0.96 (0.91, 1.02) | 82.5 | **1.07 (1.03, 1.11)** |
| 300-999 | 15.9 | **1.22 (1.09, 1.37)** | 50.4 | **1.14 (1.09, 1.20)** |  | 62.7 | 1.05 (0.99, 1.11) | 81.9 | **1.07 (1.03, 1.11)** |
| 1000 or more | 12.2 | 1 (reference) | 43.0 | 1 (reference) |  | 60.3 | 1 (reference) | 77.0 | 1 (reference) |
| Workplace smoking ban status |  |  |  |  |  |  |  |  |  |
| Complete ban | 5.4 | 1 (reference) | 28.0 | 1 (reference) |  | 41.2 | 1 (reference) | 66.4 | 1 (reference) |
| Partial ban | 18.0 | **2.47 (2.11, 2.87)** | 56.2 | **1.74 (1.64, 1.85)** |  | 59.9 | **1.35 (1.24, 1.47)** | 82.1 | **1.19 (1.13, 1.25)** |
| No ban | 36.1 | **3.98 (3.39, 4.67)** | 72.8 | **1.94 (1.82, 2.06)** |  | 65.0 | **1.52 (1.39, 1.66)** | 89.2 | **1.26 (1.20, 1.33)** |

CI, confidence interval; NA, not applicable; SHS, secondhand smoke.

^a^Adjusted for listed all variables.

Boldface indicates statistical significance (p < 0.05).
