# Supplementary material for: Disparity and Trends in Secondhand Smoke Exposure among Japanese Employees, Particularly Smokers vs. Non-Smokers
Source: PLoS One. 2016 Apr 6;11(4):e0152096. doi: 10.1371/journal.pone.0152096 (PMC4822844; doi:10.1371/journal.pone.0152096)
Supplement: S3 Table — (DOCX) [file pone.0152096.s003.docx]

**Table S3. Trends in prevalence and rate ratio for everyday workplace SHS exposure from other people among employees according to characteristics (unweighted results)**

|  | **2002** | | **2007** | | **2012** | |
| --- | --- | --- | --- | --- | --- | --- |
|  | *Everyday SHS exposure (%)* | *Rate ratio^a^ (95% CI)* | *Everyday SHS exposure (%)* | *Rate ratio^a^ (95% CI)* | *Everyday SHS exposure (%)* | *Rate ratio^a^ (95% CI)* |
|  |  |  |  |  |  |  |
| **Total** | 42.7 | NA | 28.5 | NA | 21.6 | NA |
| **Smoking status** |  |  |  |  |  |  |
| *Nonsmoker* | 30.2 | 1 (reference) | 15.3 | 1 (reference) | 9.5 | 1 (reference) |
| *Smoker* | 63.3 | **1.97 (1.88, 2.06)** | 58.6 | **3.47 (3.25, 3.70)** | 53.6 | **4.90 (4.44, 5.41)** |
| **Sex** |  |  |  |  |  |  |
| *Men* | 48.0 | 1 (reference) | 34.3 | 1 (reference) | 27.0 | 1 (reference) |
| *Women* | 32.7 | **0.93 (0.88, 0.98)** | 19.0 | 0.96 (0.89, 1.03) | 11.9 | **0.78 (0.69, 0.89)** |
| **Age group** |  |  |  |  |  |  |
| *Less than 30 years* | 48.7 | 1 (reference) | 32.8 | 1 (reference) | 25.3 | 1 (reference) |
| *30-39 years* | 43.3 | **0.86 (0.82, 0.90)** | 30.6 | **0.92 (0.86, 0.98)** | 23.8 | **0.84 (0.74, 0.95)** |
| *40-49 years* | 40.9 | **0.82 (0.78, 0.87)** | 27.3 | **0.82 (0.77, 0.88)** | 20.8 | **0.76 (0.67, 0.86)** |
| *50-59 years* | 40.8 | **0.82 (0.78, 0.86)** | 25.7 | **0.80 (0.74, 0.86)** | 17.8 | **0.64 (0.55, 0.74)** |
| *60 years or more* | 29.6 | **0.63 (0.55, 0.73)** | 19.1 | **0.67 (0.57, 0.79)** | 17.1 | **0.68 (0.54, 0.84)** |
| **Employment category** |  |  |  |  |  |  |
| *Regular employee* | 44.3 | **1.12 (1.04, 1.21)** | 30.2 | **1.09 (1.00, 1.20)** | 22.6 | 0.95 (0.82, 1.09) |
| *Others, including part-time worker* | 32.0 | 1 (reference) | 20.3 | 1 (reference) | 17.2 | 1 (reference) |
| **Worksite scale (number of employees)** |  |  |  |  |  |  |
| *10-29* | 42.0 | **0.90 (0.83, 0.98)** | 35.3 | **1.31 (1.17, 1.48)** | 24.8 | 1.19 (1.00, 1.42) |
| *30-49* | 47.0 | 0.99 (0.91, 1.07) | 30.6 | **1.24 (1.10, 1.40)** | 26.2 | **1.29 (1.07, 1.55)** |
| *50-99* | 43.2 | 0.97 (0.90, 1.05) | 32.5 | **1.37 (1.22, 1.53)** | 25.0 | **1.22 (1.02, 1.46)** |
| *100-299* | 42.7 | 0.99 (0.92, 1.07) | 28.3 | **1.24 (1.11, 1.39)** | 21.1 | 1.09 (0.91, 1.30) |
| *300-999* | 42.2 | 1.05 (0.98, 1.13) | 25.9 | **1.27 (1.13, 1.42)** | 18.1 | 0.99 (0.83, 1.17) |
| *1000 or more* | 39.0 | 1 (reference) | 19.4 | 1 (reference) | 17.1 | 1 (reference) |
| **Workplace smoking ban status** |  |  |  |  |  |  |
| *Complete ban* | 14.1 | 1 (reference) | 10.7 | 1 (reference) | 13.4 | 1 (reference) |
| *Partial ban* | 40.5 | **2.40 (1.73, 3.34)** | 28.7 | **2.10 (1.76, 2.50)** | 22.9 | **1.50 (1.34, 1.69)** |
| *No ban* | 53.7 | **2.98 (2.14, 4.14)** | 43.3 | **2.41 (2.01, 2.89)** | 38.1 | **2.13 (1.83, 2.47)** |

CI, confidence interval; NA, not applicable; SHS, secondhand smoke

^a^Adjusted for all listed variables

Boldface indicates statistical significance (p <0.05).
