# Supplementary material for: Disparity and Trends in Secondhand Smoke Exposure among Japanese Employees, Particularly Smokers vs. Non-Smokers
Source: PLoS One. 2016 Apr 6;11(4):e0152096. doi: 10.1371/journal.pone.0152096 (PMC4822844; doi:10.1371/journal.pone.0152096)
Supplement: S4 Table — (DOCX) [file pone.0152096.s004.docx]

**Table S4. Trends in prevalence and rate ratio for workplace SHS exposure from other people (everyday or sometimes) among employees according to characteristics (unweighted results)**

|  | **2002** | | **2007** | | **2012** | |
| --- | --- | --- | --- | --- | --- | --- |
|  | *Everyday or sometimes SHS exposure (%)* | *Rate ratio^a^*  *(95% CI)* | *Everyday or sometimes SHS exposure (%)* | *Rate ratio^a^*  *(95% CI)* | *Everyday or sometimes SHS exposure (%)* | *Rate ratio^a^*  *(95% CI)* |
|  |  |  |  |  |  |  |
| **Total** | 77.0 | NA | 60.5 | NA | 49.6 | NA |
| **Smoking status** |  |  |  |  |  |  |
| *Nonsmoker* | 71.4 | 1 (reference) | 51.5 | 1 (reference) | 39.6 | 1 (reference) |
| *Smoker* | 86.4 | **1.14 (1.12, 1.16)** | 81.1 | **1.41 (1.37, 1.45)** | 76.1 | **1.72 (1.62, 1.83)** |
| **Sex** |  |  |  |  |  |  |
| *Men* | 81.1 | 1 (reference) | 66.5 | 1 (reference) | 56.0 | 1 (reference) |
| *Women* | 69.4 | **0.94 (0.92, 0.96)** | 50.7 | **0.93 (0.90, 0.96)** | 38.2 | **0.84 (0.78, 0.90)** |
| **Age group** |  |  |  |  |  |  |
| *Less than 30 years* | 80.5 | 1 (reference) | 64.6 | 1 (reference) | 54.6 | 1 (reference) |
| *30-39 years* | 76.3 | **0.94 (0.92, 0.96)** | 62.4 | **0.96 (0.94, 0.99)** | 53.0 | **0.92 (0.85, 1.00)** |
| *40-49 years* | 76.8 | **0.96 (0.93, 0.98)** | 58.4 | **0.91 (0.88, 0.94)** | 46.7 | **0.82 (0.75, 0.89)** |
| *50-59 years* | 76.4 | **0.94 (0.91, 0.96)** | 59.2 | **0.93 (0.90, 0.96)** | 44.6 | **0.76 (0.69, 0.84)** |
| *60 years or more* | 69.9 | **0.89 (0.84, 0.94)** | 51.5 | **0.83 (0.76, 0.90)** | 49.2 | **0.88 (0.77, 1.01)** |
| **Employment category** |  |  |  |  |  |  |
| *Regular employee* | 78.9 | **1.13 (1.09, 1.18)** | 62.5 | **1.06 (1.01, 1.11)** | 51.2 | 1.03 (0.95, 1.13) |
| *Others, including part-time worker* | 64.7 | 1 (reference) | 50.7 | 1 (reference) | 43.2 | 1 (reference) |
| **Worksite scale (number of employees)** |  |  |  |  |  |  |
| *10-29* | 76.9 | **1.06 (1.01, 1.11)** | 68.0 | **1.31 (1.23, 1.39)** | 58.6 | **1.44 (1.28, 1.62)** |
| *30-49* | 82.0 | **1.09 (1.04, 1.13)** | 63.7 | **1.25 (1.18, 1.34)** | 58.4 | **1.42 (1.26, 1.60)** |
| *50-99* | 77.2 | **1.07 (1.03, 1.12)** | 66.3 | **1.32 (1.25, 1.41)** | 53.9 | **1.30 (1.16, 1.46)** |
| *100-299* | 77.7 | **1.08 (1.04, 1.13)** | 63.0 | **1.29 (1.22, 1.37)** | 49.9 | **1.22 (1.09, 1.37)** |
| *300-999* | 76.9 | **1.09 (1.05, 1.13)** | 55.7 | **1.19 (1.11, 1.26)** | 42.3 | 1.04 (0.93, 1.16) |
| *1000 or more* | 70.2 | 1 (reference) | 47.2 | 1 (reference) | 38.8 | 1 (reference) |
| **Workplace smoking ban status** |  |  |  |  |  |  |
| *Complete ban* | 35.7 | 1 (reference) | 32.9 | 1 (reference) | 37.1 | 1 (reference) |
| *Partial ban* | 76.2 | **2.04 (1.70, 2.44)** | 61.6 | **1.74 (1.59, 1.90)** | 52.1 | **1.39 (1.29, 1.49)** |
| *No ban* | 84.0 | **2.17 (1.81, 2.60)** | 76.6 | **1.88 (1.71, 2.06)** | 71.2 | **1.63 (1.47, 1.80)** |

CI, confidence interval; NA, not applicable; SHS, secondhand smoke

^a^Adjusted for all listed variables

Boldface indicates statistical significance (p <0.05).
